# Regulation of Dual-Specificity Phosphatase (DUSP) Ubiquitination and Protein Stability

**DOI:** 10.3390/ijms20112668

**Published:** 2019-05-30

**Authors:** Hsueh-Fen Chen, Huai-Chia Chuang, Tse-Hua Tan

**Affiliations:** Immunology Research Center, National Health Research Institutes, Zhunan 35053, Taiwan; 061013@nhri.edu.tw

**Keywords:** dual-specificity phosphatase, mitogen-activated protein kinase, ubiquitination, protein stability

## Abstract

Mitogen-activated protein kinases (MAPKs) are key regulators of signal transduction and cell responses. Abnormalities in MAPKs are associated with multiple diseases. Dual-specificity phosphatases (DUSPs) dephosphorylate many key signaling molecules, including MAPKs, leading to the regulation of duration, magnitude, or spatiotemporal profiles of MAPK activities. Hence, DUSPs need to be properly controlled. Protein post-translational modifications, such as ubiquitination, phosphorylation, methylation, and acetylation, play important roles in the regulation of protein stability and activity. Ubiquitination is critical for controlling protein degradation, activation, and interaction. For DUSPs, ubiquitination induces degradation of eight DUSPs, namely, DUSP1, DUSP4, DUSP5, DUSP6, DUSP7, DUSP8, DUSP9, and DUSP16. In addition, protein stability of DUSP2 and DUSP10 is enhanced by phosphorylation. Methylation-induced ubiquitination of DUSP14 stimulates its phosphatase activity. In this review, we summarize the knowledge of the regulation of DUSP stability and ubiquitination through post-translational modifications.

## 1. The DUSP Family Phosphatases

Mitogen-activated protein kinases (MAPKs) are the important components of cell signaling pathways. MAPKs regulate physiological and pathological responses to various extracellular stimuli and environmental stresses [1,2,3,4,5,6,7]. The best-known members of the MAPK family are ERK, JNK, and p38 subgroups [5,6]. These kinases unusually require dual phosphorylation on both threonine and tyrosine residues within the conserved motif T-X-Y for kinase activity [8]. The MAPK signaling pathways are involved in the processes of gene transcription, mRNA translation, protein stability, protein localization, and enzyme activity, thus regulating various cellular functions including cell proliferation, cell differentiation, cell survival, and cell death [9,10]. MAPK signaling pathways are also involved in a number of diseases including inflammation and cancer [11,12]. Pathway outputs reflect the balance between the activation of upstream pathways and the inhibition of negative regulators. Inactivation of MAPKs are mediated by serine/threonine phosphatases, tyrosine phosphatase, and dual-specificity phosphatases (DUSPs) through dephosphorylation of threonine and/or tyrosine residues of the T-X-Y motif within the kinase activation loop [13]. The largest group of protein phosphatases that specifically regulates the MAPK activity in mammalian cells is the DUSP family phosphatases [13].

The DUSP family phosphatases dephosphorylate both threonine/serine and tyrosine residues of their substrates. All DUSPs have a common phosphatase domain, which contains conserved Asp, Cys, and Arg residues forming the catalytic site. A subfamily of DUSPs contains the MAP kinase-binding (MKB) motif or the kinase-interacting motif (KIM) that interacts with the common docking domain of MAPKs to mediate the enzyme–substrate interaction [14,15]. DUSPs containing the KIM domain are generally classified as typical DUSPs or MAP kinase phosphatases (MKPs), whereas DUSPs without the KIM domain are generally classified as atypical DUSPs (Table 1). However, there are a few exceptions. Three KIM-containing typical DUSPs, namely, DUSP2 (PAC1), DUSP5, and DUSP8, are not named as MKPs (Table 1). Two atypical DUSPs, DUSP14 (MKP6) and DUSP26 (MKP8), do not contain the KIM domain but still can dephosphorylate and inactivate MAPKs (Table 1). Typical DUSPs can be further grouped into three subgroups based on their predominant subcellular locations, that is, the nucleus, the cytoplasm, or both [15]. 

DUSPs do not always require phosphatase activity to regulate the function of substrates. For example, DUSPs can control functions of MAPKs by sequestering them in the cytoplasm or nucleus [16,17,18]. Because both DUSPs and substrates of MAPKs interact with MAPKs via the common docking domain of MAPKs [19], DUSPs may also regulate MAPK signaling by competing with MAPK substrates for binding to MAPKs [15].

DUSPs are critical for the regulation of MAPK activity and are thus subject to complex regulation. Gene expression and phosphatase activity of DUSPs are regulated by gene transcription, protein modification, or protein stability. This review will focus on the regulation of DUSP protein stability and ubiquitination by post-translational modifications. 

## 2. Negative Regulation of DUSPs by Lys48-Linked Ubiquitination and Proteasomal Degradation 

Ubiquitination regulates many biological functions such as cell proliferation, cell apoptosis, and immune responses [20]. Ubiquitination is the modification of a protein by ubiquitin(s) on one or more lysine residues. Ubiquitination is mediated by an enzyme cascade involving three classes of enzymes: E1 (ubiquitin-activating enzyme), E2 (ubiquitin-conjugating enzyme), and E3 (ubiquitin ligase), resulting in covalent bonding of ubiquitin to lysine residues of protein substrates [21]. Ubiquitin contains seven lysine residues (Lys6, 11, 27, 29, 33, 48, and 63) that can act as ubiquitin acceptor forming ubiquitin chains with different topologies on protein substrates. The functions of Lys48-linked and Lys63-linked ubiquitinations are well characterized. Lys48-linked ubiquitination primarily controls proteasomal degradation; Lys63-linked ubiquitination controls several protein functions, including receptor endocytosis, protein trafficking, enzyme activity, and protein–protein interaction [22]. In addition, Lys48- and Lys63-linked ubiquitinations are associated with lysosomal degradation [23]. 

DUSP1 (MKP1) is a nuclear phosphatase [24]. DUSP1 binds to JNK and p38 with stronger affinity compared to its binding to ERK, leading to their dephosphorylation and inactivation [25]. Reciprocally, ERK induces DUSP1 proteasomal degradation by enhancing nuclear translocation and transcription activity of the transcription factor forkhead box M1 (FoxM1) [26], leading to the induction of the ubiquitin E3 ligase complex S-phase kinase-associated protein (Skp2)/cyclin-dependent kinase regulatory subunit 1 (Cks1) [27,28,29,30]. Besides ERK, other signaling molecules also control DUSP1 degradation. DUSP1 underwent an ubiquitin-mediated proteasomal degradation in response to glutamate-induced oxidative stress [31]. The involved E3-ubiquitin ligase was not identified; however, the process depends on the presence of PKCδ [31]. EGF plus lactoferrin induce a rapid proteasomal degradation of DUSP1, resulting in sustained ERK activation in human fibrosarcoma [32]. In rat cardiac myoblast H9c2 cells, the ubiquitin E3 ligase Atrogin-1 interacts with DUSP1 and promotes the ubiquitin-mediated proteasomal degradation of DUSP1, thereby leading to sustained activation of JNK signaling and subsequent cell apoptosis and ischemia/reperfusion injury [33]. Conversely, ubiquitin-specific peptidase 49 (USP49; a deubiquitinase) interacts with and deubiquitinates DUSP1, resulting in DUSP1 stabilization [34]. Angiotensin II-stimulated proteasome activity results in DUSP1 degradation and subsequent STAT1 activation in T cells, leading to induction of Th1 differentiation [35]; however, it is unclear how DUSP1 is regulated by angiotensin II. DUSP1 knockdown results in prolonged and enhanced STAT1 phosphorylation; it remains unclear whether DUSP1 can directly dephosphorylate STAT1.

DUSP4 (MKP2) preferentially inhibits ERK and JNK [36]. In senescent human fibroblasts, the phosphatase activity and protein levels of DUSP4 are increased due to impaired proteasomal activity [37]. 8-Bromo-cAMP (8-Br-cAMP) stimulation leads to reduction of the proteasomal degradation of DUSP4 in Leydig cells [38]; DUSP4 stabilization results in inhibition of ERK activity and subsequent reduction of the synthesis of P450scc steroidogenic enzyme, which is critical for steroid synthesis [38]. 

DUSP5 displays phosphatase activity toward ERK; DUSP5 overexpression results in both inactivation and nuclear translocation of ERK [16]. DUSP5 is a short-lived protein, which is ubiquitinated and subjected to proteasomal degradation [39]. DUSP5 degradation enhances the amplitude and duration of ERK signaling [40]. Reciprocally, ERK induces DUSP5 stability by decreasing DUSP5 ubiquitination [39]; the regulation is independent of ERK kinase activity but dependent on ERK–DUSP5 interaction [39].

DUSP6 (MKP3) preferentially inhibits ERK [41,42]. Reduction of DUSP6 by reactive oxygen species (ROS) is correlated with high ERK activity [43]. Similarly, in thyrocytes, B-Raf (V600E) mutation induces ROS generation, leading to proteasomal degradation of DUSP6 [44]. The B-Raf (V600E)-induced DUSP6 degradation results in ERK activation and cell senescence [44]. The anti-diabetic drug metformin accelerates the development of B-Raf (V600E)-mediated melanoma by inducing proteasomal degradation of DUSP6 through AMPK [45]. In breast cancer cells, PKCδ depletion results in ERK activation by inducing the level of the ubiquitin E3 ligase Nedd4, which induces DUSP6 degradation [46]. In contrast, thyroid-stimulating hormone (TSH) stabilizes DUSP6 by enhancing the expression of manganese superoxide dismutase (MnSOD), leading to prevention of senescence and induction of papillary thyroid carcinoma [44]. 

DUSP7, an ERK phosphatase, is ubiquitinated under hypoxic stress [47]. Hypoxia-inducible factors (HIFs) induce expression and cytoplasmic accumulation of the ubiquitin E3 ligase speckle-type POZ protein (SPOP) in clear cell renal cell carcinoma under hypoxic stress [47]. SPOP induces tumorigenesis by promoting ubiquitination and degradation of multiple regulators, including DUSP7 [47].

DUSP8 (M3/6) preferentially inactivates JNK and maybe p38 [48,49,50]. The protein synthesis inhibitor anisomycin [51] enhances the JNK pathway via activation of its upstream kinase SEK/MKK4 [52]. Anisomycin also stimulates JNK activity by inducing ubiquitination and degradation of DUSP8 [52]. In contrast, the proteasome inhibitor lactacystin prevents DUSP8 degradation, resulting in dephosphorylation and inactivation of JNK [52]. 

DUSP9 (MKP4), an ERK phosphatase, is associated with maintenance of the stemness of embryonic stem cells (ESCs) [53]. The long non-coding RNA (lncRNA) LincU directly binds and protects the DUSP9 protein from ubiquitin-mediated proteasomal degradation; the stabilized DUSP9 inhibits ERK activation, leading to preservation of naïve pluripotency of ESCs [53].

DUSP16 is also regulated by ubiquitin-mediated proteasomal degradation, and their ubiquitinations are regulated by phosphorylation (see below). In addition to Lys48-linked ubiquitination, one DUSP (DUSP14) is regulated by Lys63-linked ubiquitination (see Section 3.3).

## 3. Other Post-Translational Regulations of DUSP Ubiquitination and/or Stability 

### 3.1. Phosphorylation

DUSP1 stability is differentially regulated by sustained or transient activation of ERK. Sustained activation of ERK phosphorylates DUSP1 on Ser296 and Ser323 residues [54]. The Ser296/323 phosphorylation of DUSP1 facilitates its interaction with the ubiquitin E3 ligase CUL1/SKP2/CKS1 complex, which targets DUSP1 for proteasomal degradation [27,54,55]. In contrast, ERK reduces DUSP1 degradation by phosphorylating two other residues, Ser359 and Ser364 [56,57]. Transient activation of ERK stimulates DUSP1 Ser359/364 phosphorylation, which enhances DUSP1 stability, and feedback attenuates ERK signaling [56]. One group reported that this ERK-induced DUSP1 stabilization may be independent of ubiquitination [57]. Furthermore, Krüpple-like transcription factor 5 (KLF5) promotes breast cancer cell survival partially through ERK-induced DUSP1 Ser359/364 phosphorylation, which is essential for DUSP1 protein stabilization [58].

DUSP1 stabilization results in inhibition of JNK and p38 activation, as well as subsequent inhibition of TNF-α and IL-6 production [59]. Glucocorticoids prevent animals from autoimmune diseases due to enhancement of DUSP1 expression and stability [60,61,62]. Insulin stimulation enhances DUSP1 phosphorylation, resulting in DUSP1 stabilization in vascular smooth muscle cells (VSMCs) [63]. This increase of DUSP1 leads to reduction of ERK activity and subsequent inhibition of cell migration [63]. The phosphorylated calcium/calmodulin kinase II (CaMKII) interacts with DUSP1 and prevents DUSP1 from proteasomal degradation [64]. Conversely, dephosphorylation of CaMKII leads to disruption of CaMKII–DUSP1 interaction, leading to proteasomal degradation of DUSP1 [64]. 

DUSP2 stability is induced by the atypical MAP kinase ERK4 [65]. Wild-type ERK4, but not catalytically inactive ERK4, binds to and stabilizes DUSP2 proteins [65]. This finding suggests that DUSP2 may be phosphorylated by ERK4, leading to DUSP2 stabilization.

DUSP4 is rapidly induced after ERK activation [66]. ERK interacts with and phosphorylates DUSP4 on Ser386 and Ser391 residues within the C-terminus [67], leading to prevention of DUSP4 from ubiquitin-mediated proteasomal degradation. The mechanism provides a negative feedback control of ERK activity [67,68]. Consistently, a short spliced isoform (encoding 303 amino acids) of human DUSP4 lacking the MAP kinase binding site is more susceptible to ubiquitination and proteasomal degradation than that of DUSP4 [69]. It is noted that one group reported no detectable effect of ERK on DUSP4 ubiquitination [57].

DUSP6 can also be a substrate of ERK. Upon serum stimulation, ERK phosphorylates DUSP6 on Ser159 and Ser197 residues in fibroblast cells [70]. The ERK-induced DUSP6 phosphorylation triggers ubiquitination and proteasomal degradation of DUSP6 [70]. EGF plus lactoferrin induce proteasomal degradation of DUSP6 [32]. P2X7 nucleotide or EGF also stimulates DUSP6 Ser197 phosphorylation by ERK, resulting in proteasomal degradation of DUSP6 in neurons and astrocytes [71]. Thus, ERK exerts a positive-feedback mechanism on its own kinase activity by promoting the degradation of DUSP6 [70,71]. In addition, insulin induces DUSP6 degradation through the ERK-mediated DUSP6 Ser159/197 phosphorylation in liver cells [72]. The reduction of DUSP6 by insulin signaling leads to downregulation of glucose-6-phosphatase, resulting in inhibition of glucose output of liver cells [72]. In addition to ERK-mediated degradation of DUSP6, mTOR signaling also induces the phosphorylation of DUSP6 on Ser159 residue and its subsequent proteasomal degradation [73]. Intracellular reactive oxygen species (ROS) accumulation such as hydrogen peroxide causes DUSP6 phosphorylation on Ser159 and Ser197 residues, leading to ubiquitination and degradation of DUSP6 in ovarian cancer cells [43].

DUSP6 is ubiquitinated and degraded by proteasome in the early phase of platelet-derived growth factor-B chains (PDGF-BB) stimulation; the process requires MEK-induced phosphorylation of DUSP6 on Ser174 residue [74]. In the later phase, DUSP6 is induced by ERK-mediated transcriptional expression, leading to inhibition of ERK activity [74]. Interestingly, both protein degradation and mRNA synthesis of DUSP6 are ERK-dependent, indicating both positive and negative regulation of DUSP6 by ERK [74]. Therefore, the regulation of DUSP6 by PDGF-BB stimulation exhibits a negative feedback control of PDGF-BB signaling [74]. 

DUSP10 is phosphorylated by mTORC2 on Ser224 and Ser230 residues upon insulin stimulation, leading to stabilization of DUSP10 and subsequent inactivation of p38 in glioblastoma cells [75].

DUSP16 preferentially inactivates JNK [17] and maybe p38 [76]. ERK phosphorylates Ser446 residue of DUSP16, resulting in enhancement of DUSP16 protein stability [77]. DUSP16 protein levels are rapidly decreased by ubiquitination and subsequent proteasomal degradation in quiescent cells [77]. ERK also phosphorylates DUSP16 on Ser446 residue [77,78]. This phosphorylation leads to stabilization of DUSP16 by preventing ubiquitination [77]. Induction of DUSP16 strongly suppresses JNK activation. Therefore, the activation of the ERK pathway can strongly inhibit JNK activation by stabilizing DUSP16 [77,78]. 

### 3.2. Oxidation 

DUSP1 and DUSP4 proteins can be oxidized under oxidative stress. Oxidation of catalytic cysteine within the active site of DUSPs inactivates DUSP phosphatase activities and triggers their proteasomal degradation [55,79]. Superoxide induces DUSP1 proteasomal degradation, leading to JNK activation and subsequent cell death in lung cancer cells [80]. Metabolic disorder-induced oxidative stress causes DUSP1 S-glutathionylation and subsequent proteasomal degradation of DUSP1, resulting in monocyte migration and macrophage recruitment [81]. In addition, oxidation of DUSP1 induces its proteasomal degradation in monocytes upon asbestos stimulation, leading to induction of p38 activity and TNFα gene expression [82]. 

DUSP4 is redox sensitive. Under cadmium ion (Cd^2+^)-induced oxidative stress, DUSP4 is oxidized by glutathione disulfide (GSSG), leading to DUSP4 degradation and cell apoptosis [83]. In contrast, N-acetylcysteine (NAC) treatment upregulates the protein levels of DUSP4, protecting cells from cadmium ion (Cd^2+^)-induced apoptosis [83].

### 3.3. Methylation

**Figure 1:**
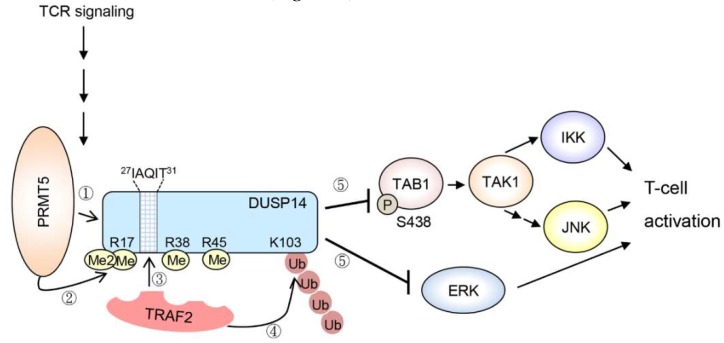
Upon T-cell receptor (TCR) signaling, the protein arginine methyltransferase PRMT5 interacts with DUSP14 and induces its methylation on Arg17, Arg38, and Arg45 residues. Arginine-methylated DUSP14 then interacts with the ubiquitin E3 ligase TRAF2, which binds to the motif containing IAQIT residues of DUSP14 and then promotes K63-linked ubiquitination on Lys103 residue of DUSP14. Methylation and subsequent ubiquitination enhance the phosphatase activity of DUSP14. Activated DUSP14 dephosphorylates TAB1, leading to sequential inactivation of TAK1 and downstream IKK/JNK activities. Activated DUSP14 also directly dephosphorylates ERK and attenuates the ERK signaling pathway. Arrows denote activation; T bars denote inhibition.

DUSP14 (MKP6) is a MAP kinase phosphatase that inactivates JNK, ERK, and p38 in vitro [84]. DUSP14 is a negative regulator of T-cell receptor (TCR) signaling by directly inhibiting ERK in T cells [84]. DUSP14 also attenuates T-cell activation by directly dephosphorylating TAB1, leading to inhibition of TAB1–TAK1 complex and its downstream signaling molecules JNK and IKK [85]. Upon TCR signaling, DUSP14 interacts with the ubiquitin E3 ligase TRAF2, which promotes Lys63-linked ubiquitination on Lys103 residue of DUSP14 [86]. DUSP14 Lys63-linked ubiquitination is induced by methylation [87]. During TCR signaling, protein arginine methyltransferase 5 (PRMT5) interacts with DUSP14 and triggers its methylation on Arg17, Arg38, and Arg45 residues [87]. DUSP14 contains a TRAF2-binding motif, ^27^IAQIT^31^, which is adjacent to these methylation sites. DUSP14 methylation results in recruitment of the ubiquitin E3 ligase TRAF2, which in turn induces Lys63-linked ubiquitination on Lys103 residue of DUSP14 [86,87]. Methylation and subsequent ubiquitination stimulate the phosphatase activity of DUSP14. Taken together, methylation-induced ubiquitination of DUSP14 promotes the activation of DUSP14 phosphatase activity during TCR signaling, resulting in attenuation of T-cell activation [85,86,87] (Figure 1).

## 4. Dysregulation of DUSPs in Diseases

DUSPs are involved in immune cell homeostasis, inflammatory responses, metabolic regulation, and cancer development/progression [15,88]. For example, DUSP6 knockout mice show impaired T-cell glycolysis and increased T follicular helper cell (T_FH_) differentiation [89]. DUSP6 knockout mice also show altered gut microbiome and transcriptome response against diet-induced obesity [90]. Moreover, DUSP6 downregulation is correlated with cancer progression of human pancreatic adenocarcinoma and lung cancer [91,92]. DUSP2 downregulation induces colon cancer stemness [93]. DUSP3 downregulation occurs in human non-small cell lung cancer patients [94]; consistently, DUSP3 deficiency results in enhanced cancer cell migration [95]. DUSP5 is also downregulated in human gastric and colorectal cancers [96,97]. In addition, DUSP22 knockout mice show enhanced T-cell-mediated immune responses and are more susceptible to experimental autoimmune encephalomyelitis (EAE) [98]; consistently, DUSP22 protein levels are decreased in T cells of human systemic lupus erythematosus (SLE) patients [99]. DUSP22 expression is also downregulated in human T-cell lymphoma [100,101]. Therefore, further studies of DUSP protein stability and/or ubiquitination may help understand the complex interplay between cell signaling pathways and disease pathogenesis.

## 5. Conclusions

**Figure 2:**
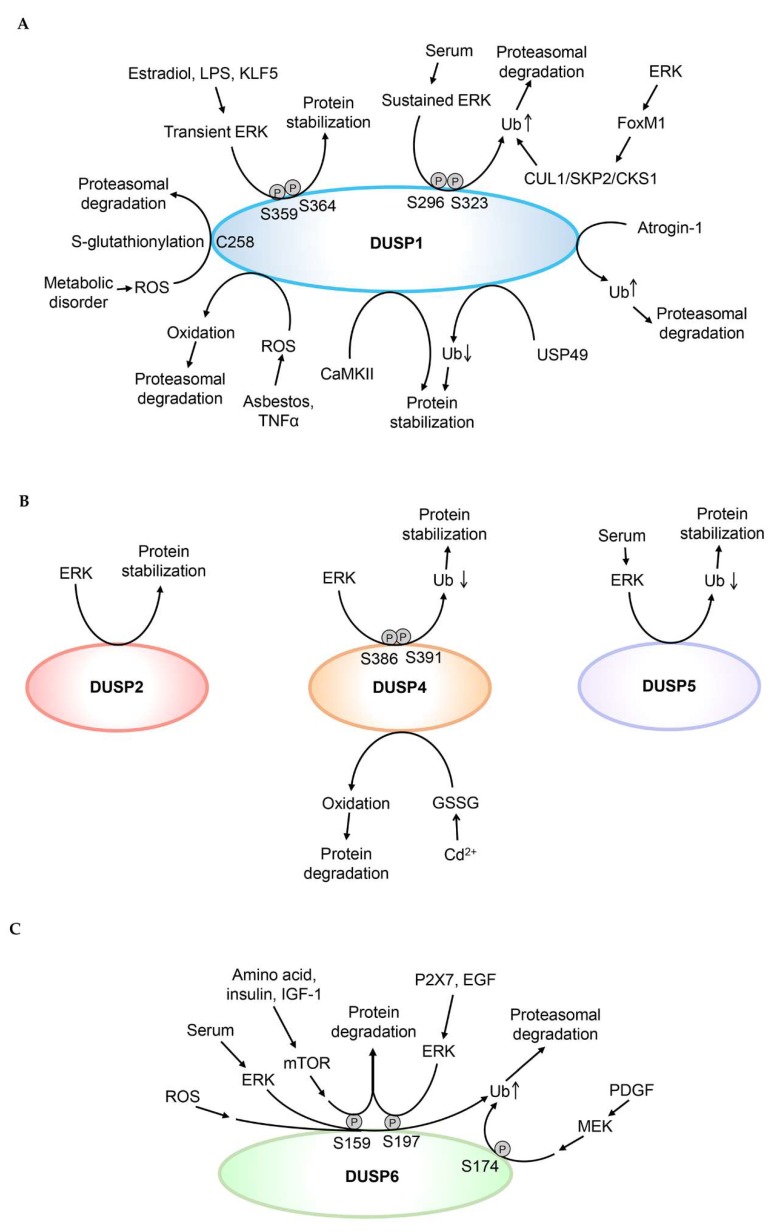

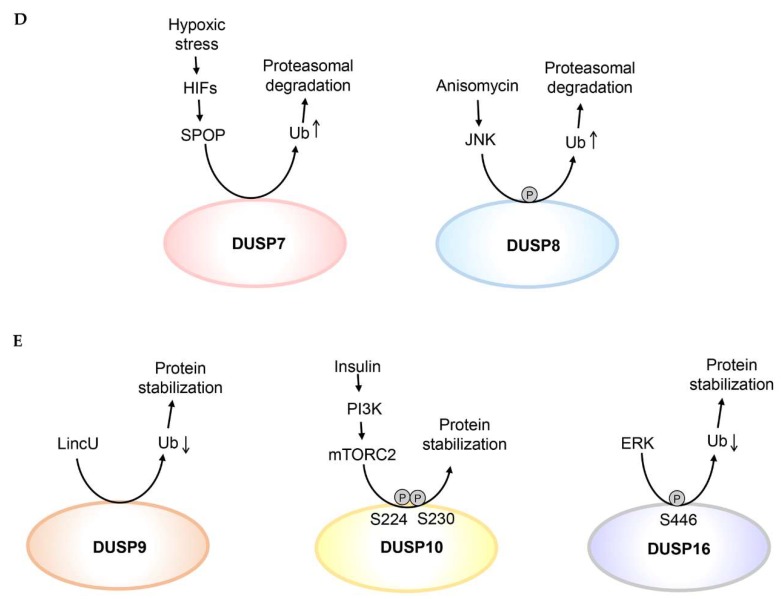
Post-translational modifications regulate DUSP protein stability. (**A**) Ubiquitination, oxidation, Cys258 S-glutathionylation, or Ser296/Ser323 phosphorylation of DUSP1 induces DUSP1 proteasomal degradation. Deubiquitination or phosphorylation of Ser359 and Ser364 residues enhances DUSP1 protein stability. (**B**) ERK induces DUSP2 protein stabilization; however, it is unclear whether ERK directly phosphorylates DUSP2. Phosphorylation of Ser386 and Ser391 residues of DUSP4 enhances its protein stability by inhibiting ubiquitin-mediated proteasomal degradation. Oxidation induces DUSP4 protein degradation. Reduced ubiquitination of DUSP5 enhances its protein stability. (**C**) Phosphorylation of Ser159, Ser174, or Ser197 residue induces proteasomal degradation of DUSP6. (**D**) Ubiquitination of DUSP7 or DUSP8 induces their proteasomal degradation. (**E**) Reduced ubiquitination of DUSP9 or DUSP16 enhances their protein stability. Phosphorylation of Ser224 and Ser230 residues enhances DUSP10 protein stability. Ub denotes ubiquitination of DUSPs. Oxidation indicates oxidation of DUSP1 or DUSP4.

The DUSP family phosphatases are key regulators of MAPK activity [13]. Because the half-lives of many DUSPs are only about 1 h, protein levels of DUSPs are tightly regulated by post-translational modifications [15]. The post-translational regulations of DUSP proteins are summarized in Figure 2. The studies for protein stability of DUSPs are summarized in Table 2.

The protein stability of DUSP1, DUSP4, DUSP5, DUSP6, DUSP7, DUSP8, DUSP9, and DUSP16 are regulated by ubiquitin-mediated proteasomal degradation. Protein stability of DUSP2 and DUSP10 is increased by ERK and mTORC2, respectively [65,75]; however, it is unclear whether ubiquitination is involved in the degradation of these two phosphatases. To date, only three ubiquitin E3 ligases and one deubiquitinase (also named ubiquitin-specific peptidase (USP)) for degradation of DUSPs have been identified. The ubiquitin E3 ligases CUL1 and Atrogin-1 are responsible for DUSP1 ubiquitination; the ubiquitin E3 ligase SPOP is responsible for DUSP7 ubiquitination [33,47,54]. The deubiquitinase for DUSP1 is USP49 [34]. Additional ubiquitin E3 ligases and deubiquitinases for controlling proteasomal degradation of DUSPs await to be identified. The ubiquitination/proteasomal degradation of DUSPs are usually regulated by phosphorylation. Although MAPKs are dephosphorylated by DUSPs, MAPKs also reciprocally control protein stability of DUSPs and their downstream signaling pathways. One major kinase for DUSPs is ERK. Transient activation of ERK phosphorylates DUSP1 on Ser359 and Ser364 residues to stabilize DUSP1, providing a negative feedback that attenuates ERK activity [56]. In contrast, sustained ERK activity induces DUSP1 Ser296/323 phosphorylation and subsequent protein degradation, resulting in further enhancement of ERK signaling [27,54]. Consistently, both sustained ERK activation and decreased DUSP1 protein levels are observed in cancer cells [102,103]. Moreover, ERK phosphorylates DUSP6 on Ser159 and Ser197 residues, facilitating DUSP6 proteasomal degradation [70]. The MEK/ERK pathway also mediates DUSP6 Ser174 phosphorylation and subsequently induces DUSP6 degradation [74]. Unlike the inhibitory effect of ERK on DUSP6, ERK increases DUSP4 protein stability by phosphorylating DUSP4 on Ser386 and Ser391 residues [57]. Enhancement of DUSP4 protein levels leads to inhibition of ERK activity. Similarly, ERK protects DUSP16 from proteasomal degradation by phosphorylating DUSP16 on Ser446 residue [77]. DUSP16 preferentially dephosphorylates JNK and maybe p38 compared to ERK [17,76], suggesting that ERK-mediated DUSP16 induction negatively regulates the activation of JNK or maybe p38. Besides ERK, mTOR also regulates protein stability of DUSPs. mTORC2 phosphorylates DUSP10 on Ser224 and Ser230 residues and subsequently enhances DUSP10 protein stability, leading to reduction of p38 activity [75]. Moreover, mTOR signaling also induces DUSP6 Ser159 phosphorylation, resulting in proteasomal degradation of DUSP6 [73]. 

In addition to phosphorylation, oxidation of DUSPs also induces protein degradation. Asbestos or TNFα stimulation generates ROS that oxidizes DUSP1 and induces DUSP1 proteasomal degradation [79,82]. Cd^2+^-induced oxidative stress triggers DUSP4 oxidation by glutathione disulfide (GSSG), resulting in protein degradation of DUSP4 [83]. 

Ubiquitination not only controls protein stability but also induces protein activity. The ubiquitin E3 ligase TRAF2-mediated Lys63-linked ubiquitination of DUSP14 enhances its phosphatase activity. It would be interesting to study whether Lys63-linked ubiquitination also regulates the activity of other DUSPs.

To our knowledge, eight DUSPs (DUSP1, DUSP4, DUSP5, DUSP6, DUSP7, DUSP8, DUSP9, and DUSP16) are known to be degraded by the proteasome, and it is important to understand whether other members of the DUSP family are also regulated by ubiquitin-mediated proteasomal degradation. Lastly, it would be useful to study whether other modifications, such as sumoylation, neddylation, acetylation, and glycosylation also control DUSP ubiquitination and protein stability. 

## Figures and Tables

**Table 1:** Classification and domain structure of human dual-specificity phosphatases (DUSPs).

Classification	Gene Symbol	Alias	Domain Structure		MAPK Substrates	
Typical DUSPs (also named MKPs)	DUSP1	MKP1, CL100, VH1, HVH1, PTPN10	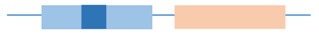		JNK, p38 > ERK	
	DUSP4	MKP2, VH2, HVH2, TYP	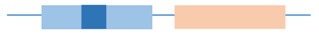		ERK, JNK > p38	
	DUSP6	MKP3, PYST1	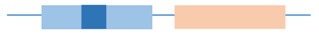		ERK	
	DUSP7	PYST2, MKPX^*^	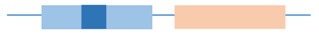		ERK	
	DUSP9	MKP4	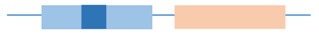		ERK > p38	
	DUSP10	MKP5	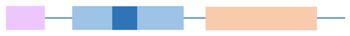		JNK, p38	
	DUSP16	MKP7	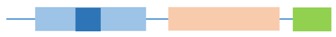		JNK (p38?)	
Typical DUSPs (not named as MKPs)	DUSP2	PAC1	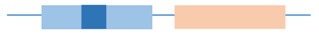		ERK, JNK, p38	
	DUSP5	VH3, HVH3	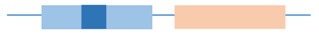		ERK	
	DUSP8	HB5, VH5, HVH-5, HVH8, (Mouse: M3/6)	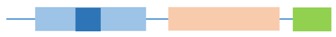		JNK (p38?)	
Atypical DUSPs	DUSP3	VHR	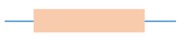			
	DUSP11	PIR1	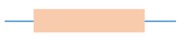			
	DUSP12	YVH1	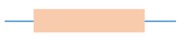			
	DUSP13	DUSP13A, DUSP13B, BEDP, MDSP, SKRP4, TMDP	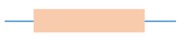			
	DUSP15	VHY	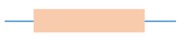			
	DUSP18	DUSP20, LMW-DSP20	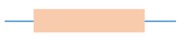			
	DUSP19	DUSP17, LMW-DSP3, SKRP1, TS-DSP1	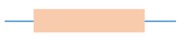			
	DUSP21	LMW-DSP21	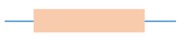			
	DUSP22	JKAP, JSP1, VHX, LMW-DSP2, MKPX^*^	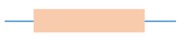			
	DUSP23	DUSP25, VHZ, LDP-3, MOSP	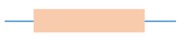			
	DUSP24	STYXL1, MK-STYX	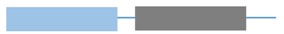			
	DUSP27		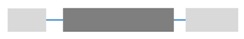			
	DUSP28	VHP, DUSP26^#^	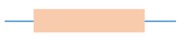			
Atypical DUSPs (also named MKPs)	DUSP14	MKP6, MKP-L	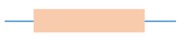		JNK > ERK > p38	
	DUSP26	MKP8, LDP-4, NATA1, SKRP3, NEAP, DUSP24^#^	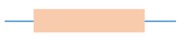		p38 (ERK?)	
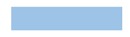	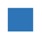	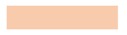	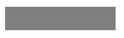	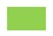	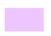	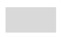
Cdc25-homology	Kinase-interacting motif (KIM)	Phosphatase	Phosphatase (inactive)	PEST	Disintegrin	Unknown

^*^, MKPX is a duplicate name for both DUSP7 and DUSP22. ^#^, DUSP24 and DUSP26 are renamed to DUSP26 and DUSP28, respectively. Domain structures are annotated from the Ensemble database.

**Table 2:** Regulation of DUSP protein stability or phosphatase activity.

	Stimuli	Stability	Modification	Modification Enzyme	Experimental Methods				Reference
					Protein Level	Half-Life	Ubiquitination	Proteasome Inhibitor	
DUSP1	Serum	↓	Phosphorylation↑ (human Ser296 ^†^/Ser323 ^†^); Ubiquitination↑	ERK; CUL1	✓	✓	✓	LLnL; MG132	[28,54]
	Estradiol	↑	Phosphorylation↑ (human Ser359 ^†^/Ser364 ^†^)	ERK	✓	✓	✓	LLnL; MG132; Lactacystin	[56]
	LPS	↑	Phosphorylation↑ (human Ser359 ^†^/Ser364 ^†^)	ERK	✓	✓	?	MG132; PS-341	[57]
	Pb^2+^	↓	Ubiquitination↑		✓	✓	✓	LLnL; MG132	[27]
	Glutamate/ PKCδ	↓	Ubiquitination↑		✓		✓	MG132; LLnL; Lactacystin	[31]
	Atrogin-1 upregulation	↓	Ubiquitination↑	Atrogin-1	✓	✓	✓	MG132	[33]
	USP49 upregulation	↑	Ubiquitination**↓**	USP49	✓		✓	MG132	[34]
	KLF5 upregulation	↑	Phosphorylation↑ (human Ser359 ^†^/Ser364 ^†^)	ERK	✓	✓		MG132	[58]
	LPS	↑	Phosphorylation↑ (human Ser359 ^†^/Ser364 ^†^)	ERK	✓	✓			[59]
	Insulin	↑	Phosphorylation↑		✓			MG132; Lactacystin	[63]
	Asbestos/ ROS	↓	Oxidation↑		✓			MG132	[82]
	TNFα/ ROS	↓	Oxidation↑		✓			MG132	[79]
	ROS	↓	S-glutathionylation↑ (human Cys258 ^†^)		✓			MG132	[81]
	Glucocorticoid	↑			✓			MG132; LLnL	[60]
	EGF plus Lactoferrin	↓			✓			MG132	[32]
	Angiotensin II/ PKA	↓			✓			Bortezomib	[35]
	CaMKII inhibition	↓			✓			MG132	[64]
	Luteolin/ Superoxide	↓			✓	✓		MG132	[80]
DUSP2	ERK4	↑			✓	✓			[65]
DUSP4	LPS	↑	Phosphorylation↑ (human Ser386 ^†^/Ser391 ^†^)	ERK	✓	✓	?	MG132; PS-341	[57]
	ERK inhibitor	↓	Phosphorylation**↓** (human Ser386 ^†^/Ser391 ^†^); Ubiquitination↑	ERK	✓	✓	✓	MG132	[67]
	Cd^2+^ / Oxidative stress	↓	Oxidation↑	GSSG	✓				[83]
	8-Bromo-cAMP	↑			✓	✓		MG132	[38]
	Senescence	↑			✓	✓		MG132	[37]
DUSP5	ERK2 binding	↑	Ubiquitination**↓**		✓	✓	✓	MG132	[39]
DUSP6	Serum	↓	Phosphorylation↑ (human Ser159 ^†^/Ser197 ^†^) Ubiquitination↑	ERK	✓	✓	✓	LLnL; Lactacystin	[70,73]
	ROS	↓	Phosphorylation↑ (human Ser159 ^†^/Ser197 ^†^); Ubiquitination↑		✓		✓	MG132	[43]
	PDGF	↓	Phosphorylation↑ (human Ser174 ^†^); Ubiquitination↑		✓	✓	✓	MG132	[74]
	P2X7 nucleotide, EGF	↓	Phosphorylation↑ (human Ser197 ^†^)	ERK	✓	✓		MG132	[71]
	Insulin	↓	Phosphorylation↑ (human Ser159 ^†^/Ser197 ^†^)	ERK	✓	✓			[72]
	Amino acid, insulin, IGF-1/ mTOR	↓	Phosphorylation↑ (human Ser159 ^†^)		✓	✓			[73]
	PKCδ downregulation	↓			✓			MG132	[46]
	TSH	↑			✓	✓		MG132	[44]
	Metformin/ AMP-activated protein kinase	↓			✓			MG132	[45]
	EGF plus Lactoferrin	↓			✓			MG132	[32]
DUSP7	Hypoxic stress/ HIFs	↓	Ubiquitination↑	SPOP	✓		✓	MG132	[47]
DUSP8	Anisomycin	↓	Phosphorylation↑; Ubiquitination↑	JNK	✓	✓	?	Lactacystin	[52]
DUSP9	LincU upregulation	↑	Ubiquitination**↓**		✓	✓	✓	MG132	[53]
DUSP10	Insulin	↑	Phosphorylation↑ (human Ser224 ^†^/Ser230 ^†^)	mTORC2	✓	✓			[75]
DUSP14	TCR signaling	(Activity↑)	Methylation↑ (human Arg17 ^†^/Arg38 ^†^/Arg45); Ubiquitination↑ (human Lys103 ^†^)	PRMT5; TRAF2	✓		✓		[86,87]
DUSP16	ERK upregulation	↑	Phosphorylation↑ (human Ser446 ^†^); Ubiquitination**↓**	ERK	✓	✓	✓	MG132; MG115	[77,78]

^†^ denotes the amino acid residue is conserved in both human and mouse proteins. LPS denotes lipopolysaccharides. TSH denotes thyroid-stimulating hormone. GSSG denotes glutathione disulfide.

## Abbreviations

Ub	ubiquitination
DUSP	dual-specificity phosphatase
MKP	MAP kinase phosphatase
MAPK	mitogen-activated protein kinase
KIM	kinase-interacting motif
Skp2	S-phase kinase-associated protein
Cks1	cyclin-dependent kinase regulatory subunit 1
FoxM1	forkhead box M1
STAT1	signal transducer and activator of transcription 1
LncRNA	long non-coding RNA
EGF	epidermal growth factor
PRMT5	protein arginine methyltransferase 5
T_FH_	T follicular helper cell
EAE	experimental autoimmune encephalomyelitis
SLE	systemic lupus erythematosus
SPOP	Speckle-type POZ protein
PDGF-BB	platelet-derived growth factor-B chains
